# Smart data analysis V2: A user-friendly software for non-statisticians

**DOI:** 10.1371/journal.pone.0297930

**Published:** 2024-07-03

**Authors:** Jularat Chumnaul, Mohammad Sepehrifar

**Affiliations:** 1 Division of Computational Science, Faculty of Science, Prince of Songkla University, Hat Yai, Songkhla, Thailand; 2 Department of Mathematics and Statistics, Mississippi State University, Mississippi State, MS, United States of America; University of Maribor, SLOVENIA

## Abstract

Data analysis can be accurate and reliable only if the underlying assumptions of the used statistical method are validated. Any violations of these assumptions can change the outcomes and conclusions of the analysis. In this study, we developed Smart Data Analysis V2 (SDA-V2), an interactive and user-friendly web application, to assist users with limited statistical knowledge in data analysis, and it can be freely accessed at https://jularatchumnaul.shinyapps.io/SDA-V2/. SDA-V2 automatically explores and visualizes data, examines the underlying assumptions associated with the parametric test, and selects an appropriate statistical method for the given data. Furthermore, SDA-V2 can assess the quality of research instruments and determine the minimum sample size required for a meaningful study. However, while SDA-V2 is a valuable tool for simplifying statistical analysis, it does not replace the need for a fundamental understanding of statistical principles. Researchers are encouraged to combine their expertise with the software’s capabilities to achieve the most accurate and credible results.

## Introduction and preliminaries

In data analysis, verifying underlying assumptions associated with the selected statistical method is essential. For instance, in the case of the two independent samples *t*-test, one must assume normal distribution within each group and equality of variances for the outcome variable, except for the Welch *t*-test (see [1, 2] for further details). If any of these assumptions are not met, the test statistic (*t*) and its associated *p*-value may not be accurate [3]. Olsen (2003) [4] and Choi (2005) [5] observe that when the underlying assumptions of an applied statistical method are not met, data analysis can produce erroneous and unreliable conclusions.

In research fields such as education, science, and social sciences, the most common statistical tests are the one-sample *t*-test, independent samples *t*-test, paired samples *t*-test, one-way analysis of variance (ANOVA), Pearson’s correlation, linear regression, and Chi-square test of independence. To obtain valid and dependable results when employing these statistical tests, one must possess a robust understanding of the underlying assumptions associated with selected methods and the means to assess whether these assumptions hold. However, many non-statisticians engaged in research often lack this knowledge and overlook the necessity of verifying these underlying assumptions before delving into data analysis. Furthermore, they are unfamiliar with selecting the appropriate statistical method for their data if assumptions are not met. Various studies provide examples of this predicament. Plonsky and Gass (2011) [6] review 174 published papers on second language (L2) acquisition across 14 journals and two edited volumes and find that the underlying assumptions are checked in only 3% of the papers. Based on a sample of 606 papers published between 1990 and 2010 on language learning and L2 acquisition, Plonsky (2013) [7] finds that assumption checking occurred only in 17% of cases. Lindstromberg (2016) [8] notes that only 22% of 90 articles on language teaching published between 1997 and 2015 reported the verification of assumptions. A more recent review by Al-Hoorie and Vitta (2018) [9] scrutinized 150 articles published in 30 applied linguistics journals from 2016 onwards, demonstrating that 74.7% validated these assumptions. Recent findings by Hu and Plonsky (2021) [10] indicate that only 17% of 107 articles on language learning and L2 published between 2012 and 2017 checked and reported all assumptions for a given statistical method, and only 24% checked and reported at least one assumption. In social science research, for example, a study involving 30 psychology department participants (not specializing in methodology or statistics) examined 6 datasets analyzed using t-test, ANOVA, and regression, revealing that only 12% and 23% correctly checked the assumptions of normality and homogeneity of variance, respectively. Moreover, when interviewed, the participants exhibited a lack of awareness regarding assumption checking, the robustness of techniques concerning the assumptions, and how assumptions should be checked [11].

To allow inexperienced users to perform complex statistical analyses accurately, we developed an interactive and user-friendly application called Smart Data Analysis V2 (SDA-V2) available at https://jularatchumnaul.shinyapps.io/SDA-V2/. SDA-V2 is designed to automate various critical tasks, including data exploration, visualization, and the examination of the underlying assumptions associated with the parametric test. Additionally, the application is equipped to recommend the most appropriate statistical method for the specific dataset in question. Furthermore, it can be used to assess the quality of research instruments and determine the minimum sample size required for a study.

## Related work

Several statistical analysis software tools have hitherto been created to analyze data using the Shiny package of R software. However, most such tools were specifically tailored for particular research data domains. Notable examples include MEPHAS [12] (for medical and pharmaceutical data analysis), grapesAgri1 [13] (for agriculture data analysis), and FaDA [14] (for regular laboratory data analysis). Only a few Shiny-based software applications have been developed for general research data. For instance, Satyahadewi and Perdana (2020) [15] developed a web application for simple inferential statistical analysis without automatic data analysis functions, while Yasar et al. (2020) [16] created an interactive and user-friendly web application for statistical analysis, featuring some automatic test selection modes, called Statistical Analysis Software (http://biostatapps.inonu.edu.tr/IAY/). However, the usage of this software is challenging due to its non-English interface. Recently, Jiang et al. (2022) [17] developed the moreThanANOVA application (https://hanchen.shinyapps.io/moreThanANOVA/), which excels in swiftly and accurately conducting distribution, significance, and post-hoc tests. However, moreThanANOVA only covers statistical tests related to the comparison of the means among various groups. Therefore, recognizing this limitation, we developed an interactive and user-friendly application called SDA-V2. Table 1 presents a comparative overview of the statistical packages available in moreThanANOVA and SDA-V2, indicating the advantages of the proposed SDA-V2 software. Our results affirm the practicality and reliability of SDA-V2.

**Table 1:** Comparative overview of the statistical packages available in moreThanANOVA and SDA-V2.

Packages	moreThanANOVA	SDA-V2
Validity/Reliability		✓
Basic Statistics	✓	✓
One sample t-test		✓
Wilcoxon signed-rank test	✓	✓
Two independent samples t-test	✓	✓
Welch’s test	✓	✓
Wilcoxon rank sum test	✓	✓
Paired samples t-test	✓	✓
Wilcoxon match-paired signed rank test	✓	✓
One-way ANOVA	✓	✓
Welch ANOVA	✓	✓
Kruskal-Wallis test	✓	✓
Post-hoc tests	✓	✓
Pearson correlation		✓
Spearman rank correlation		✓
Simple linear regression		✓
Rank-based regression		✓
Quantile regressions		✓
Pearson’s Chi-squared test		✓
Fisher’s Exact test		✓
Pearson’s Chi-squared test with Yates’ correction		✓
Sample size calculation		✓
Monte Carlo Permutation Test	✓	

## Statistical packages developed in SDA-V2

SDA-V2 is available at https://jularatchumnaul.shinyapps.io/SDA-V2/. It is comprised of nine packages: Validity/Reliability, Basic Statistics, One Mean, Two Means, One-Way ANOVA, Correlation, Simple Regression, Association, and Sample Size. The details of each package are presented below.

- **Validity/Reliability**: It assesses the quality of research instruments, including content validity and reliability.
- **Basic Statistics**: It locates the basic statistics of quantitative variables, including measures of central tendency, position, and dispersion.
- **One Mean**: It compares the mean (or median) of a population with a specified constant or hypothesized value.
- **Two Means**: It compares the mean (or median) of two independent groups and the mean (or median) of two measurements obtained from the same subject.
- **One-Way ANOVA**: It compares the mean (or median) of two or more independent groups.
- **Correlation**: It determines relationships between pairs of continuous variables.
- **Simple Regression**: It models the linear relationship between two continuous variables. Additionally, it predicts the value of a response variable based on the value of an independent variable.
- **Association**: It determines the association between categorical variables.
- **Sample Size**: It calculates the minimum sample size required for the two-sided significance test.

## SDA-V2 implementation

Each package comprises three steps: Data entry, data analysis, and data output.

### Data entry

The input data table must be prepared in .csv format using UTF-8 encoding, as shown in the *Data Preparation* tab. Then, users must upload the prepared data table to the application through the *Data* panel and provide relevant research details such as the variables to be analyzed, type of research hypothesis (two-sided or one-sided), hypothesized value, and significance level in the *Variables and parameters* panel.

### Data analysis

The data and details entered through the SDA-V2 interface are processed on a server hosted by RStudio. First, SDA-V2 automatically explores and visualizes the data. Then, it examines the underlying assumptions associated with the parametric test and selects an appropriate statistical method. Readers can refer to S1 Appendix for more details on the data analysis step for each package.

### Data output

For Validity/Reliability, content validity and internal consistency reliability assessment results are displayed in the *Quality of Instrument Report* tab.

For Basic Statistics, data exploration and data visualization results, which include boxplots and histograms, and basic statistics (mean, median, minimum, maximum, range, standard deviation, variance, coefficient of variation, 1st quartile, 3rd quartile, and interquartile range), are presented under the *Data Visualization* and *Descriptive Statistics* tabs, respectively.

For One Mean, Two Means, One-Way ANOVA, Correlation, and Association, data exploration results, including graphical representations, and basic statistics, are presented in the *Data Visualization* and *Descriptive Statistics* tabs, respectively. Assumption checking results are displayed in the *Assumptions Checking* tab. The steps used to select the appropriate statistical method are presented as a flowchart in the Method Selection tab. Finally, the analysis results for the appropriate test are presented in the *Hypothesis Testing* tab.

For Simple Regression, data exploration results, which include graphical representations, and basic statistics are presented under the *Data Visualization* and *Descriptive Statistics* tabs, respectively. Assumption checking results for the original and transformed data are displayed in the *Assumptions Checking* and *Transformations Checking* tabs, respectively. The steps used to select the appropriate statistical model are shown as a flowchart in the *Model Selection* tab. Finally, the suitable model is presented in the *Suggestion Model* tab.

For Sample Size, the results are presented in the *Sample Size Report* tab, and scatter plots of sample size versus probability of type II error and power of a test are displayed in the *Data Visualization* tab.

**Fig 1:**
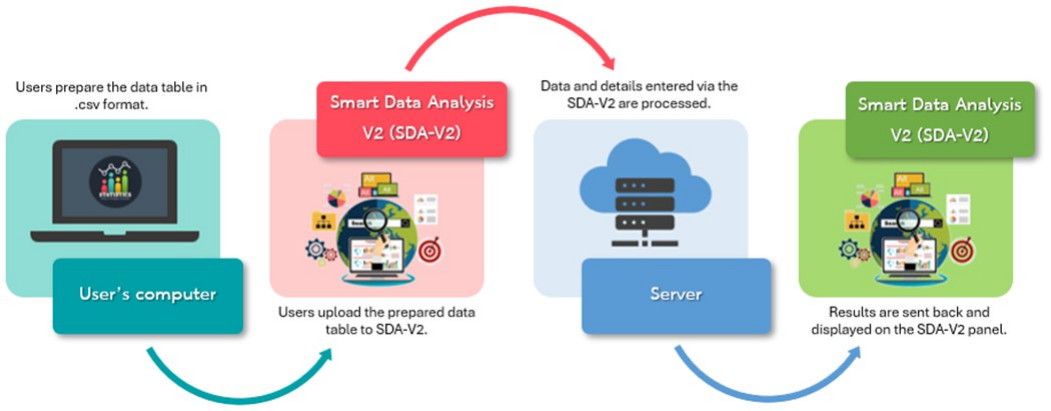
Detailed workflow. Working process of SDA-V2.

Fig 1 illustrates the working process of SDA-V2.

## SDA-V2 performance through examples

We present two examples that demonstrate SDA-V2 implementation and performance. The first example involves comparing the difference in the mean test scores between the two groups of students using two study techniques. The second example involves determining the relationship between students’ attitudes toward mathematics and mathematics achievement.

### Example I: Study techniques

A teacher wants to know whether the two study techniques result in different mean mathematics exam scores. The teacher assigns 15 students to prepare for the mathematics exam using inquiry-based learning (Group 1) and 14 students to prepare the exam using problem-based learning (Group 2). The teacher then asks each student to take the same exam, and the students’ scores are collected. For data table in .csv format, see S1 File [18].

**Fig 2:**
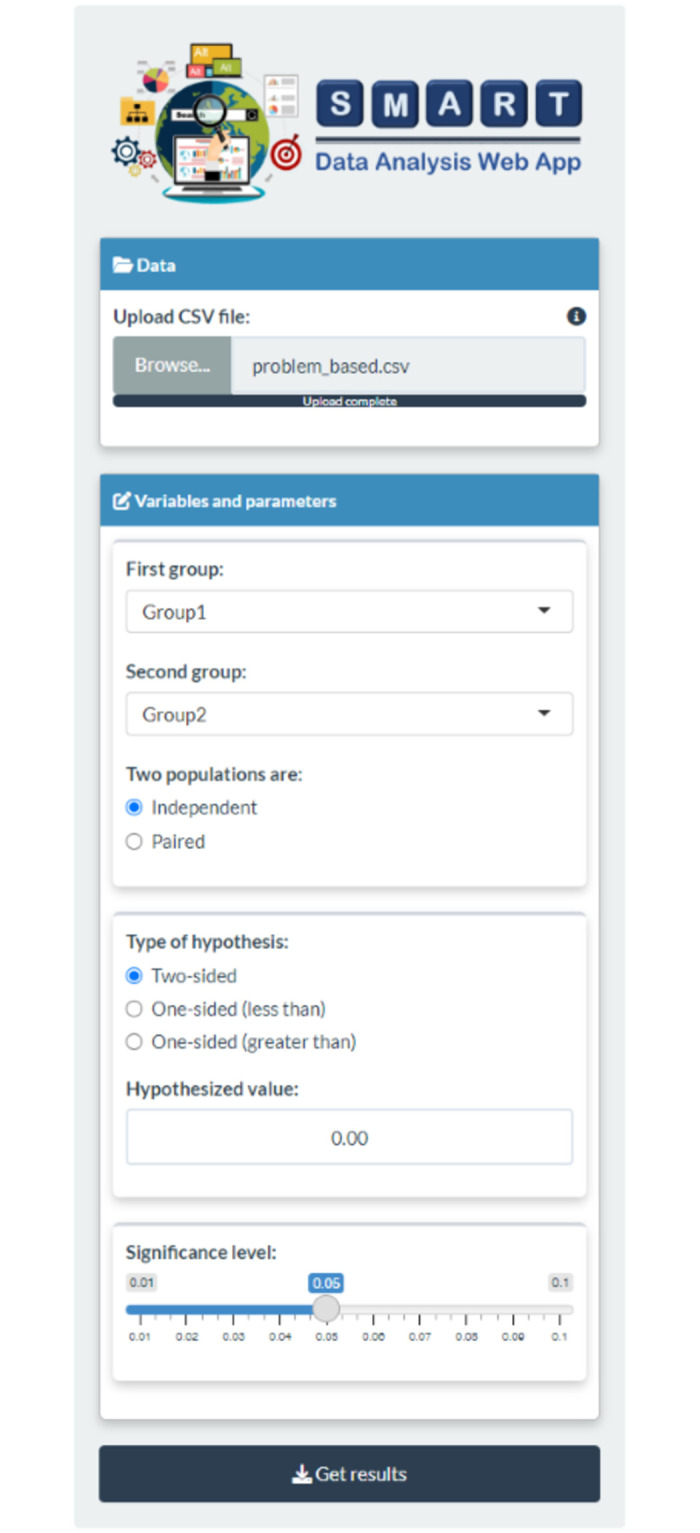
Data entry. Data table uploaded and relevant research details provided.

In this illustrative example, we apply the “Two Means” package to determine whether the mean test score differs between the two distinct groups. First, users must prepare a data table of students’exam scores in .csv format, as shown in the *Data Preparation* tab. Subsequently, the prepared data table should be uploaded to SDA-V2. After successful data upload, users have to specify the two quantitative variables, the “First group” and “Second group,” for analysis and choose the “Independence” option for the two population types. According to the research objective, users can formulate a hypothesis asserting that two different study techniques yield distinct mean mathematics exam scores. Therefore, users must choose “Two-sided” for the type of hypothesis and input “0” for the hypothesized value. Here, the significance level is set to 0.05. Fig 2 illustrates the result of the data entry step.

**Fig 3:**
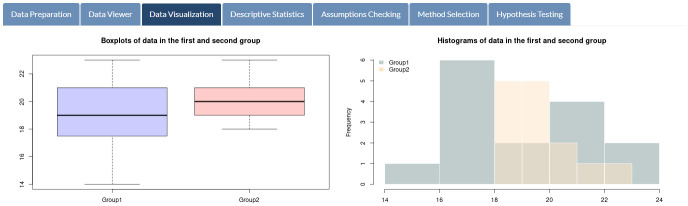
Data visualization. Boxplots and histograms of students’ mathematics exam scores.

**Fig 4:**
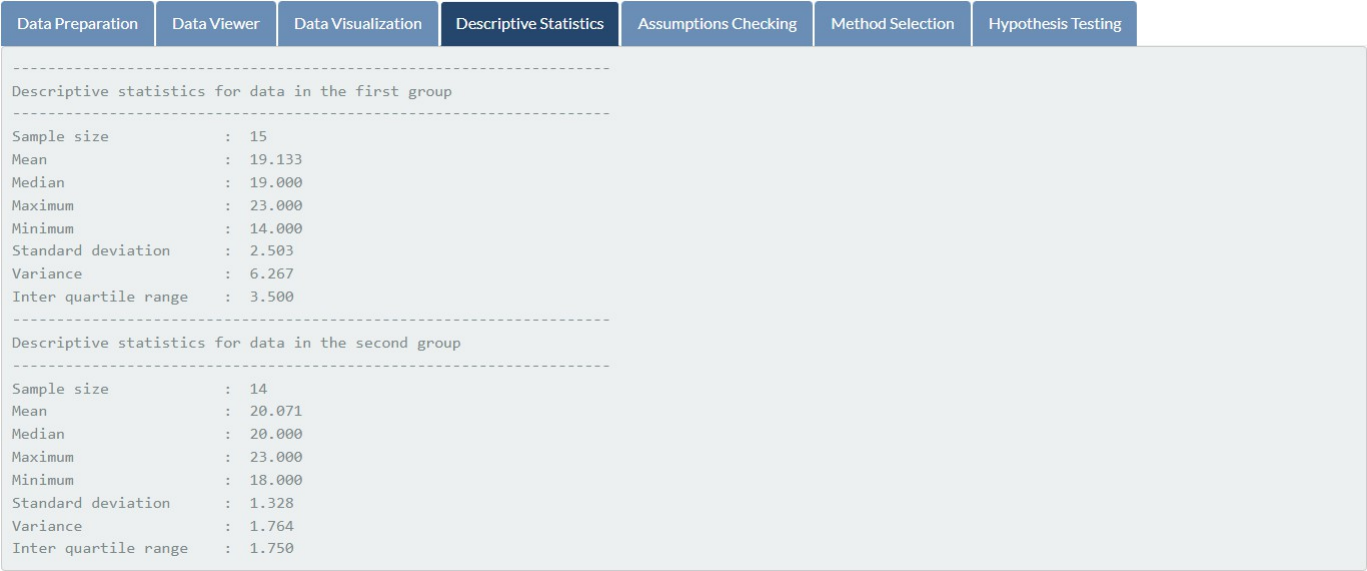
Data exploration. Basic statistics of students’ exam scores.

Once users click on the button “Get results,” all relevant analysis results are immediately displayed on SDA-V2’s tabs. Fig 3 presents boxplots and histograms of mathematics exam scores for students in both groups, while Fig 4 shows basic statistics, including sample size, mean, median, maximum, minimum, standard deviation, variance, and interquartile range of selected variables.

**Fig 5:**
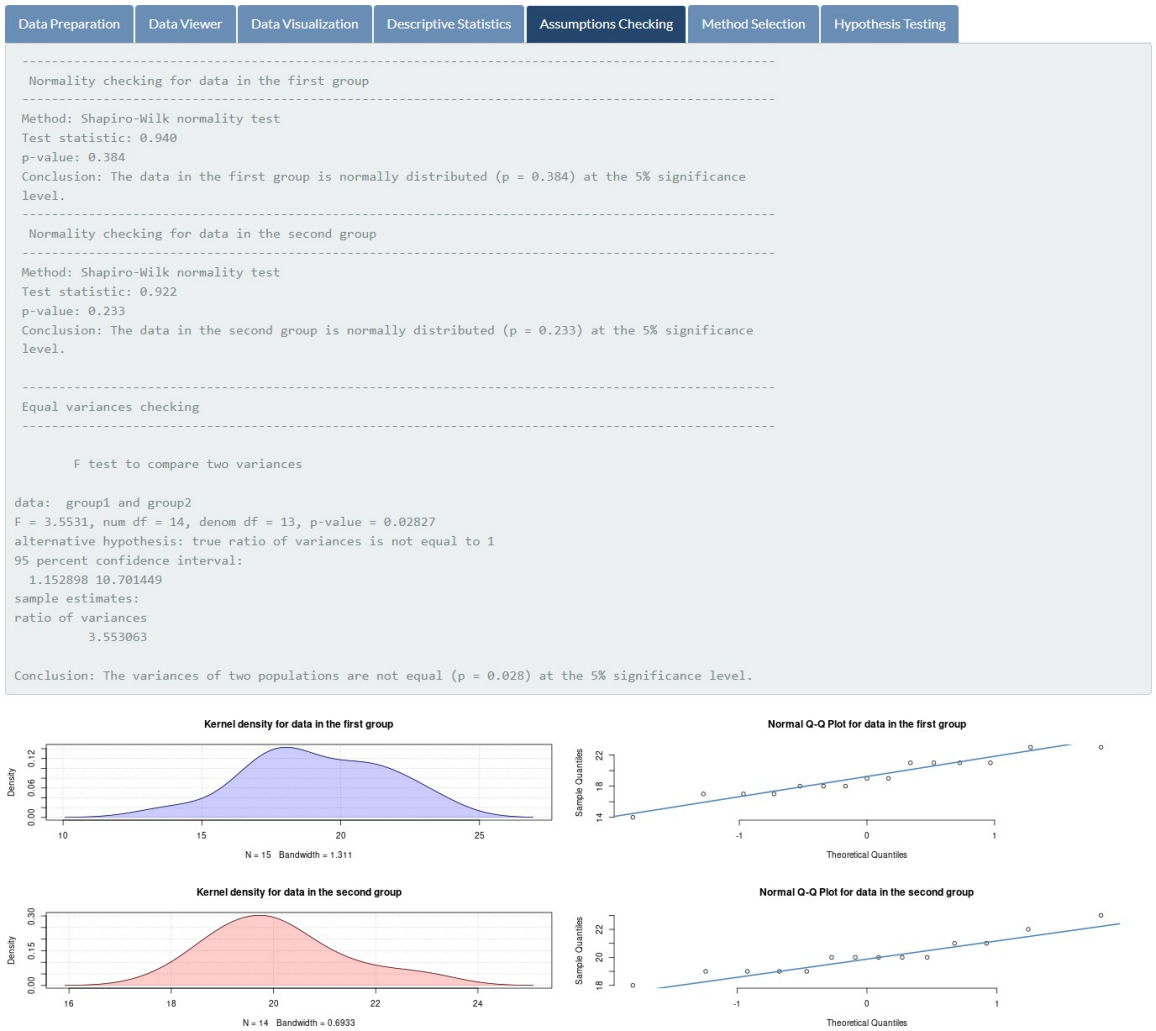
Assumptions checking. Normality and homogeneity of variance verification for students’ exam scores.

**Fig 6:**
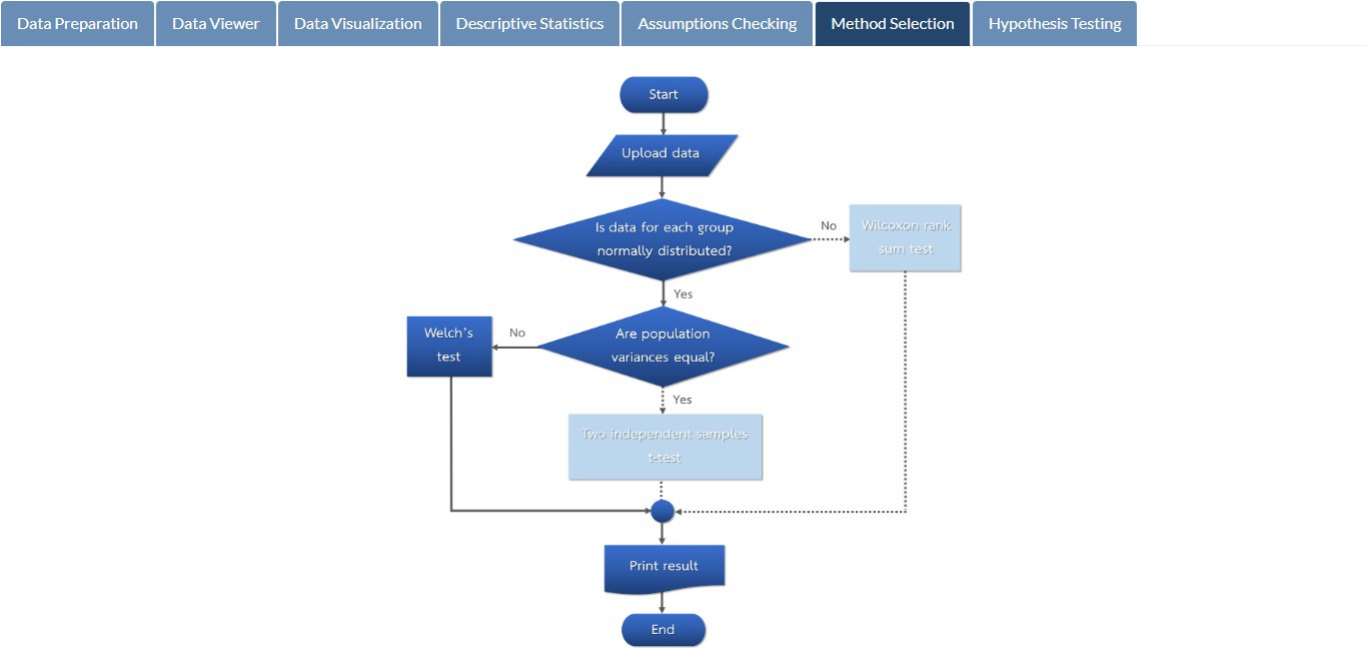
Method selection. Steps in choosing the appropriate statistical method.

**Fig 7:**
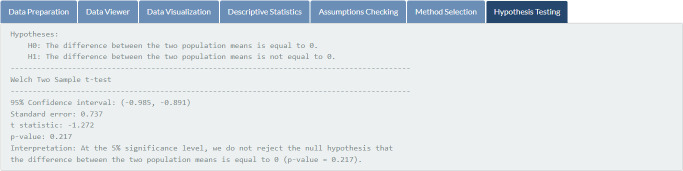
Hypothesis testing. Results of the appropriate statistical method for the input data.

Results of assumptions checking for the related parametric test (two independent samples t-test) are illustrated in Fig 5. It indicates that the mathematics exam scores of students in both groups are normally distributed (*p*-values of 0.384 and 0.233, respectively), but both variances are not equal (*p*-value = 0.028) at the 5% significance level. Therefore, the Welch test is automatically chosen and performed for the given data (see Fig 6). The hypothesis testing result based on the Welch test, as shown in Fig 7, illustrates that the two study techniques (inquiry-based and problem-based learning) do not yield different mean mathematics exam scores at the 5% significance level (*p*-value = 0.217).

### Example II: Determining a relationship between students’ attitudes and exam scores

A professor wants to examine the relationship between students’ attitudes toward mathematics and mathematics achievement. A questionnaire is presented to 38 randomly selected students, asking about their attitudes toward mathematics. Students then take the mathematics exam. For data table in .csv format, see S2 File [18].

**Fig 8:**
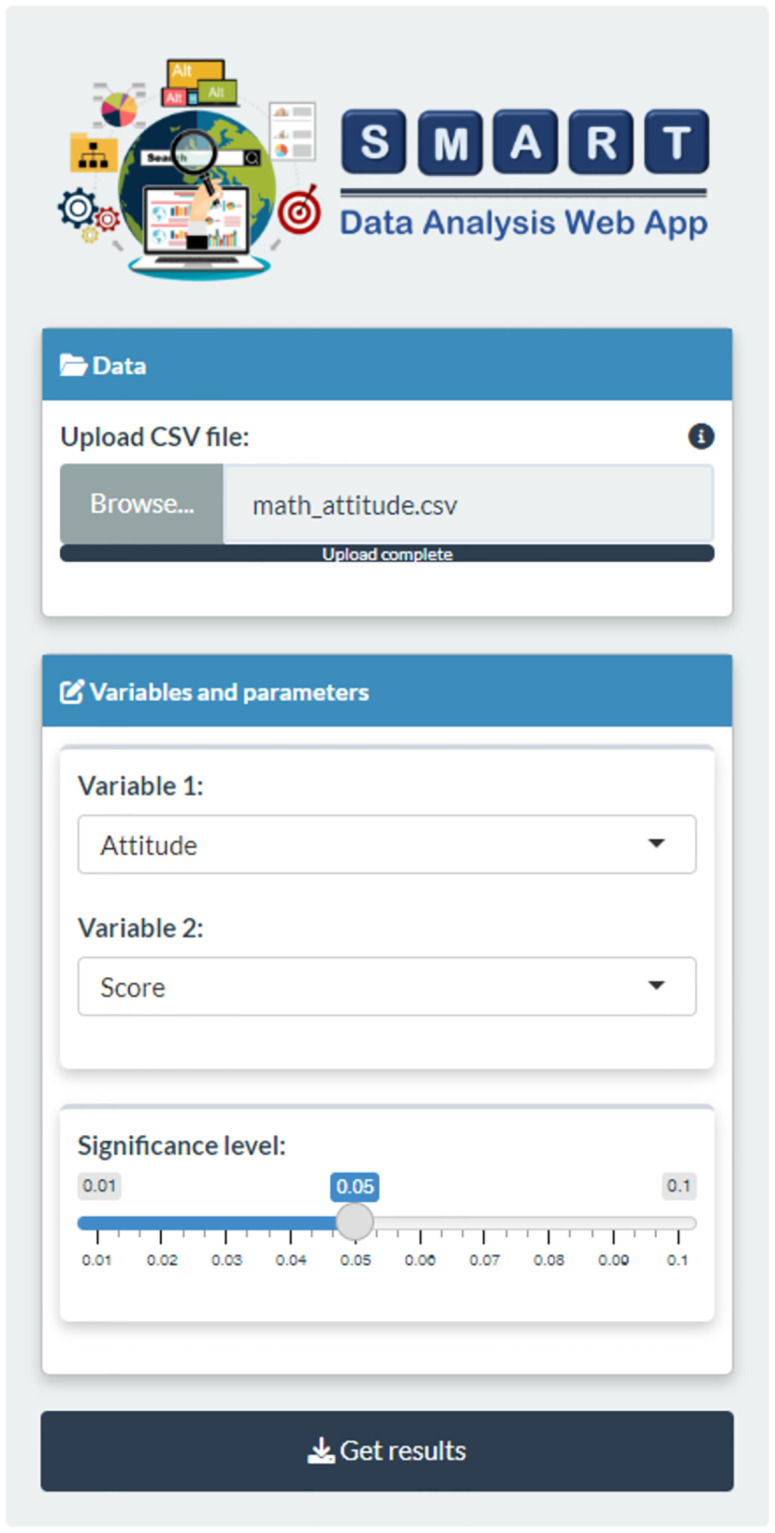
Data entry. Data table uploaded and relevant research details provided.

In this second example, we apply the “Correlation” package to determine whether a significant relationship exists between student’ attitudes toward mathematics and their mathematics achievement. Similar to Example I, users must first prepare a data table containing students’ attitudes and exam scores in .csv format and upload it to SDA-V2. Upon data upload, users must specify the two quantitative variables, “Variable 1” and “Variable 2,” for analysis and set the significance level. Fig 8 illustrates the result of the data entry step.

**Fig 9:**
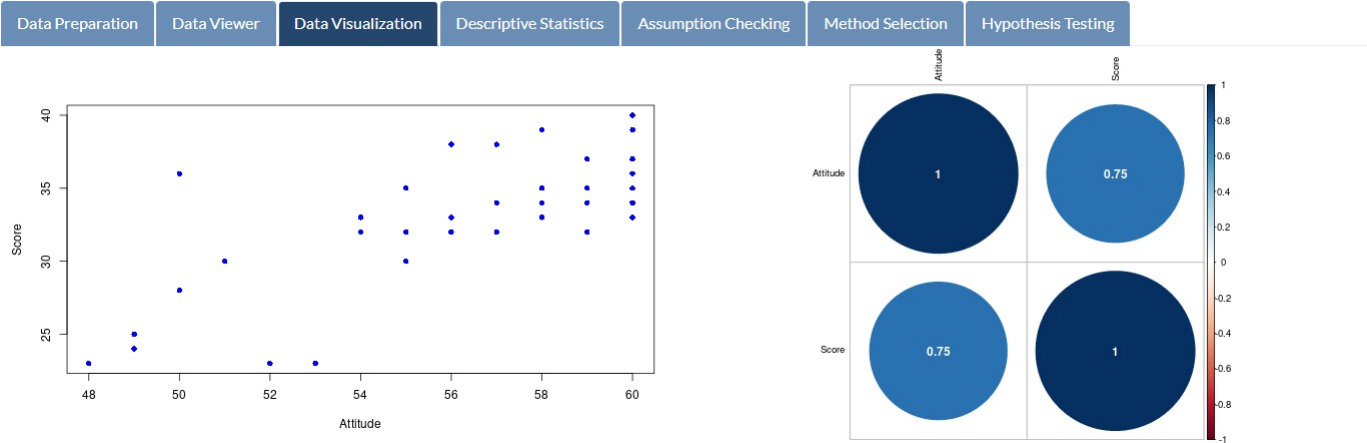
Data visualization. Scatter plot and correlogram of students’ attitudes and exam scores.

**Fig 10:**
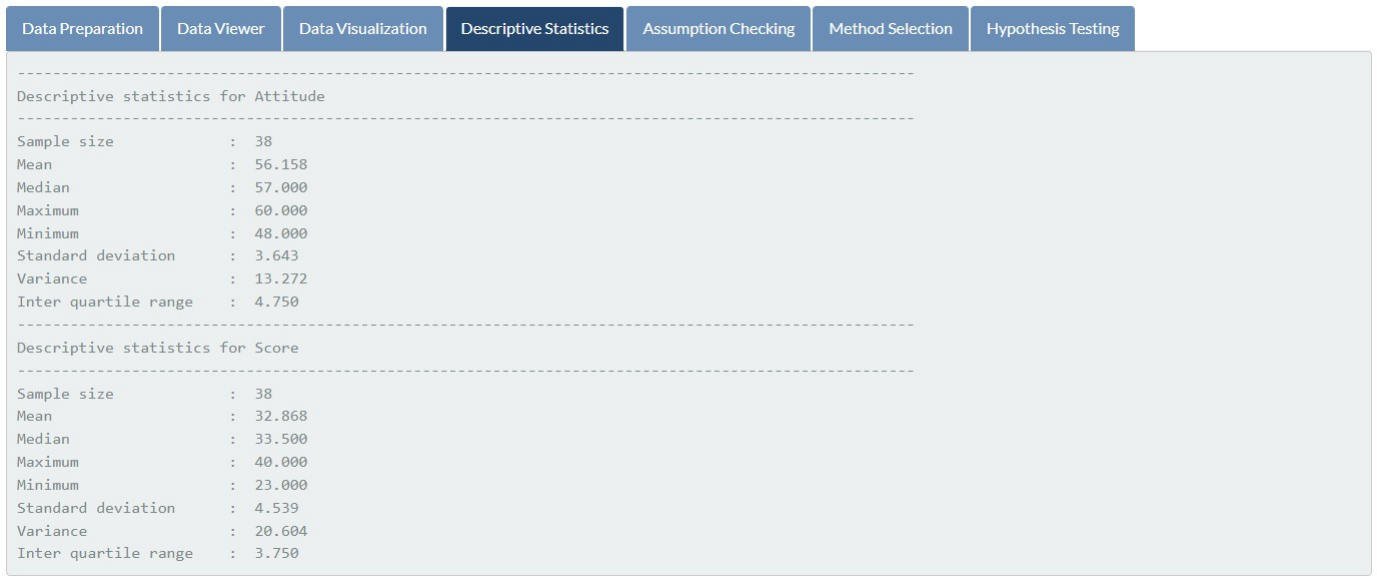
Data exploration. Basic statistics of students’ attitudes and exam scores.

**Fig 11:**
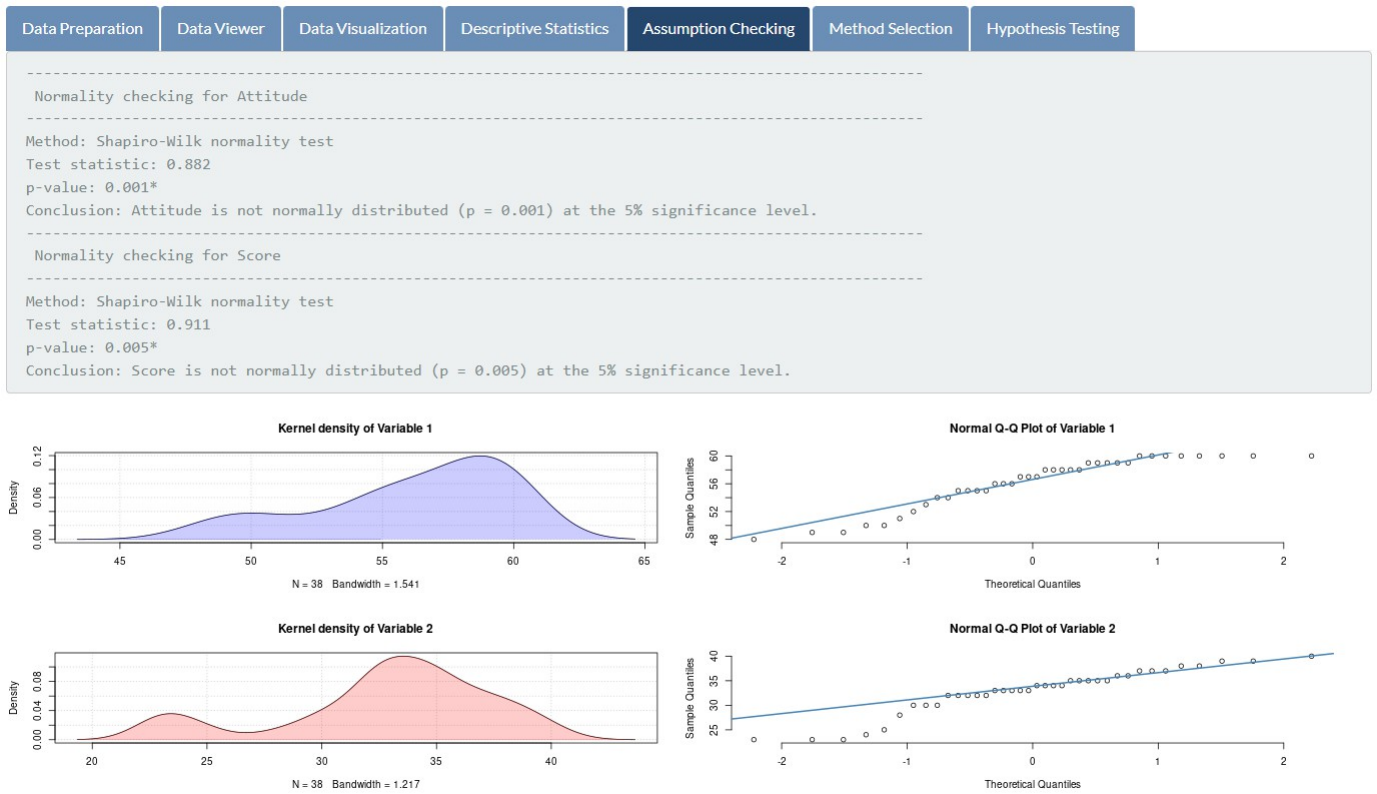
Assumptions checking. Normality checking for students’ attitudes and exam scores.

**Fig 12:**
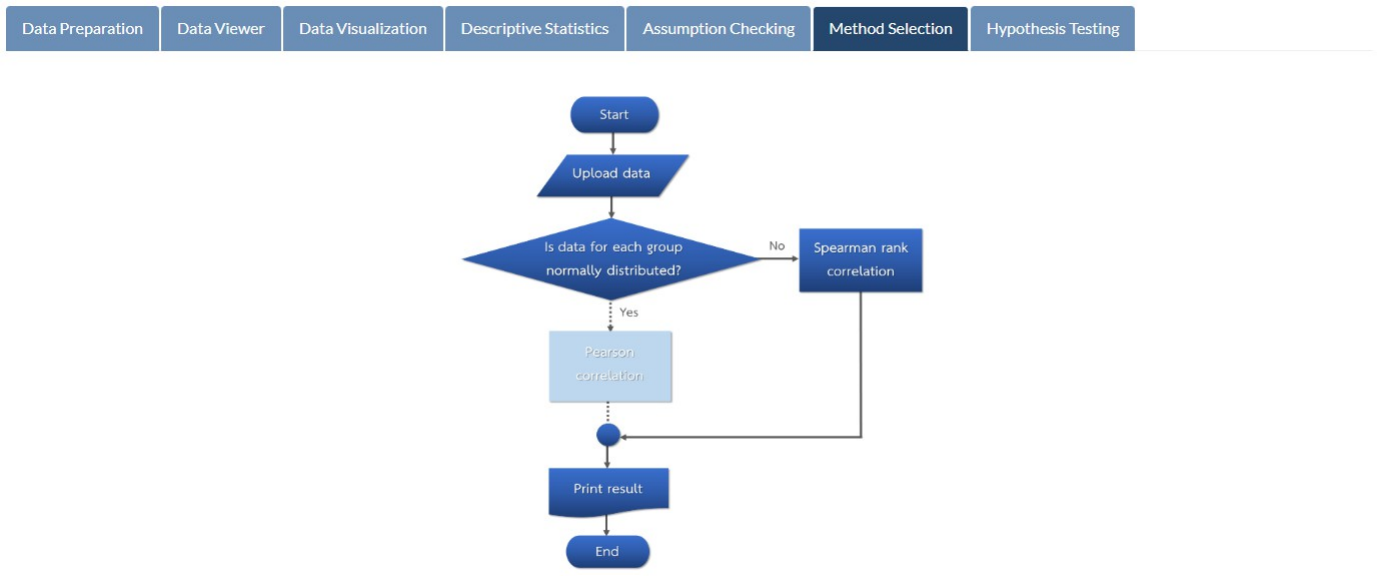
Method selection. Steps in choosing the appropriate statistical method.

**Fig 13:**
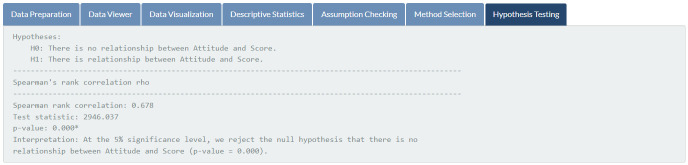
Hypothesis testing. Results of the appropriate statistical method for the input data.

After clicking the “Get results” button, all relevant analysis results are immediately displayed on SDA-V2’s tabs, as presented in Figs 9–13. Fig 9 showcases a scatter plot and correlogram depicting students’ attitudes and exam scores, while Fig 10 displays the basic statistics of selected variables.

Results of assumptions checking, featured in Fig 11, indicate that students’ attitudes toward mathematics and mathematics exam scores are not normally distributed (*p*-values of 0.001 and 0.005, respectively) at the 5% significance level. Therefore, the Spearman rank correlation is automatically chosen for the given data (see Fig 12). According to the outcome of the hypothesis testing (see Fig 13), there is sufficient evidence to support the claim that there is a relationship between students’ attitudes toward mathematics and mathematics exam scores (*p*-value < 0.001).

As demonstrated by the examples above, SDA-V2 offers a user-friendly and effective solution for non-statistician researchers. Users merely need to upload their data to SDA-V2 and provide the relevant research details. SDA-V2 then automates data exploration and visualization, examining the underlying assumptions associated with parametric tests, selecting an appropriate statistical method for the given data, and presenting analysis results in a convenient and easily comprehensible format.

## Discussion and conclusion

When using general statistical analysis software such as R, SPSS, STATA, Minitab, and Statistical Analysis System (SAS), users are typically required to possess data analysis knowledge. To obtain accurate and reliable analysis results, users must first thoroughly examine data to understand their characteristics and then select the appropriate statistics for analysis. However, many non-statistician users, such as social scientists, healthcare professionals, or students conducting research without an extensive background in statistics, must gain knowledge of assumptions and be cognizant of the necessity to verify the underlying assumptions before analyzing data. Furthermore, they often struggle with choosing the appropriate statistical method for their data when these assumptions are not met. In response to these challenges, SDA-V2 has been designed and developed to assist non-statistician users, particularly those lacking in-depth statistical knowledge, in analyzing their data more independently and efficiently. SDA-V2 offers automated data exploration, visualization, assumption checking for parametric tests, and selection of appropriate statistical methods, thus simplifying the analysis process and ensuring alignment with statistical theory. Moreover, SDA-V2 can be used to assess the quality of research instruments and determine the minimum sample size required for a study.

Compared to other well-known statistical analysis software packages, specifically Minitab and SPSS (see Table 2), although both possess user-friendly interfaces, users are still expected to have prior knowledge of the statistical methods they intend to use for conducting significance tests. They must also be aware of the underlying assumptions they must check, and understand the appropriate course of action if such assumptions are not met. For instance, when users wish to compare the means of two independent groups, the commonly used statistical method is the *t*-test. Before performing a *t*-test, users must know that they have to check the assumptions of normality and homogeneity of variances for their data, regardless of the statistical software they use. They must understand that this typically involves conducting the Shapiro–Wilk test for normality checking and the *F* test for assessing equal variances, which they cannot perform immediately with other statistical analysis software. Finally, if any of these assumptions are not met, users must know what statistical method is appropriate for their data. These processes require significant user intervention and decision-making, necessitating prior data analysis knowledge. Notably, both Minitab and SPSS are commercial software, incurring costs that can be prohibitive. Even when considering freely available statistical and data analysis software such as Statistical Lab, Statcato, SalStat Statistics, SOFA Statistics, PSPP, and OpenStat, possessing a foundational understanding of statistics is essential to analyze data. Moreover, these tools are not easy to understand and use. Free programming languages such as R and Python, while powerful and cost-effective, are much more complicated to learn and use, especially for users with no programming background.

**Table 2:** Comparison of features in SDA-V2 and well-known statistical analysis software packages (Minitab and SPSS).

Feature	SDA-V2	Minitab/SPSS
Data Import	Limited to specific formats	Supports various data formats
Data Manipulation	Basic functionalities	Robust data transformation tools
Statistical Tests	Includes common statistical tests (e.g., t-tests, ANOVA)	Comprehensive set of statistical tests with advanced options
Graphical Visualization	Basic charts	Extensive charting options
Programming Support	Limited scripting capabilities	Syntax-based programming language
User Interface	Web-based interface	GUI with point-and-click functionality
Learning Curve	User-friendly for beginners	May have a steeper learning curve
Cost	Likely free online tool	Commercial software with licensing fees
Community Support	Limited community	Large user community and support

SDA-V2 demonstrates to users how to choose the appropriate statistical method for their specific data through algorithms and flow diagrams. In statistical analysis, algorithms and flow diagrams are highlighted as valuable aids because these tools provide a systematic and structured method for choosing appropriate statistical tests. While traditional data analysis relies on a deep understanding of data types and distributions to guide the selection of statistical tests, the algorithmic approach allows researchers to utilize predefined algorithms and flow diagrams to guide the selection process. However, despite the extensive debate regarding the selection of statistical tests using algorithms and flow diagrams versus having a deep understanding of data types and distributions, these tools remain valuable aids, especially for individuals with less experience or limited expertise in statistical analysis.

In summary, SDA-V2 is designed to simplify the statistical analysis process. It is an easy-to-use and free-access web application (accessible at https://jularatchumnaul.shinyapps.io/SDA-V2/) that serves as an alternative software to assist users with limited statistical knowledge in the data analysis process rather than as a substitute for thoughtful consideration of the data.

## Supporting information

S1 Appendix Data analysis steps for each package in SDA-V2. (ZIP)

S1 File Data for Example I. Students’ exam scores.
(CSV)

S2 File Data for Example II. Students’ attitudes and exam scores.
(CSV)
