# Supplementary material for: Smart data analysis V2: A user-friendly software for non-statisticians
Source: PLoS One. 2024 Jul 3;19(7):e0297930. doi: 10.1371/journal.pone.0297930 (PMC11221694; doi:10.1371/journal.pone.0297930)
Supplement: S1 Appendix — (ZIP) [file pone.0297930.s001.zip › S1 Appendix.pdf]

Data analysis steps for each package in SDA-V2.

- **Validity/Reliability**

SDA-V2 evaluates the content validity of research instruments in terms of the Item Objective Congruence (IOC) Index and uses four methods—Split-Half, KR-20, KR-21, and Cronbach's Alpha—to determine internal consistency reliability.

Item Objective Congruence (IOC) Index

Evaluating item quality is based on the IOC index developed by Rovinelli and Hambleton (1977) [1,2]. For each item, a content validity score is determined as follows:

If the expert is confident that the item accurately measures the attribute, a score of 1 is assigned.

If the expert is confident that the item does not measure the attribute, a score of -1 is assigned.

If the expert is unsure whether the item measures the desired attribute, a score of 0 is assigned.

The IOC of each item can be computed using Eq (1):

$$IOC = \frac{\text{Total score}}{\text{Number of experts}}. \tag{1}$$

Split-half method

The Split-half method [3, 4] uses the correlation of scores obtained from two parts of a test (odd items and even items). The reliability of research instruments is computed using the split-half method as Eq (2):

$$r_t = \frac{2r_{1/2}}{1 + r_{1/2}}, \tag{2}$$

where r_t is the reliability of the entire test, and $r_{1/2}$ is the correlation with the half test.

Kuder-Richardson Formula 20 (KR-20)

The earliest and most recognized formula for assessing the internal consistency reliability of a test is KR-20 [5, 6]. The formula for dichotomous data is given by Eq (3):

$$KR20 = \frac{k}{k-1}\left[1 - \frac{\sum_{i=1}^{k} p_i(1-p_i)}{\sigma_t^2}\right], \tag{3}$$

where k is the number of items, p_i is the proportion of correct responses to test item i, and σ_t^2 is the variance of the total score.

Kuder-Richardson Formula 21 (KR-21)

Under the assumption that all items are of equal difficulty (p_i is the same for items), Kuder and Richardson used an alternative to KR-20 in the following Eq (4):

$$KR21 = \frac{k}{k-1}\left[1 - \frac{k\bar{p}(1-\bar{p})}{\sigma_t^2}\right], \tag{4}$$

where k is the number of items, $\bar{p}$ is the average correct response over all items, and σ_t^2 is the variance of the total score. This formula is known as KR-21 [5, 6].

Cronbach's Alpha Coefficient

Cronbach's Alpha Coefficient (α) [7] is a widely used method to determine the internal reliability of research instruments. Cronbach's Alpha Coefficient is defined as Eq (5):

$$\alpha = \frac{k}{k-1}\left[1 - \frac{\sum_{i=1}^{k} \sigma_i^2}{\sigma_t^2}\right], \tag{5}$$

where α is Cronbach's Alpha coefficient, k is the number of items, σ_i^2 is the variance of each item, and σ_t^2 is the variance of the total score.

- **Basic Statistics**

The "Basic Statistics" package is used to find the basic statistics of a quantitative variable, including the measures of central tendency, position, and dispersion.

- **One Mean**

For data exploration and visualization, SDA-V2 automatically creates a boxplot and histogram. Further, for any selected variable, it automatically generates commonly used basic statistics, including sample size, mean, median, maximum, minimum, standard deviation, variance, and interquartile range. SDA-V2 checks the underlying assumption associated with the one-sample t-test by automatically performing the Shapiro–Wilk normality test to determine whether data are normally distributed. This test is used because it yields higher power compared to other tests [8–10]. SDA-V2 also automatically creates a density plot and normal Q–Q plot for the selected variable, which is another technique to verify the normality assumption [11,12].

SDA-V2 automatically chooses the appropriate test for any selected variable based on the following criteria:

1. If data are normally distributed, the one sample t-test is applied [13].
2. If data are not normally distributed, a Wilcoxon signed-rank test is applied [14].

- **Two Means**

For data exploration and data visualization, if the "Independent" option in the *Variables and parameters* panel is selected, SDA-V2 automatically creates boxplots and histograms and calculates commonly used basic statistics, including sample size, mean, median, maximum, minimum, standard deviation, variance, and interquartile range for both groups. By contrast, if the "Paired" option is selected, SDA-V2 automatically creates a boxplot and histogram and finds commonly used basic statistics for the difference between the paired values.

For underlying assumptions checking, if the "Independent" option is selected, SDA-V2 automatically performs the Shapiro–Wilk normality test to verify the normal distribution of data in each group. If data in both groups are normally distributed, the application then performs the F test to compare the variances of both groups. Furthermore, SDA-V2 automatically creates density and normal Q–Q plots for data in both groups. Conversely, if the "Paired" option is selected, SDA-V2 automatically performs the Shapiro–Wilk normality test to examine if the difference between the paired values is normally distributed. It also creates a density plot and normal Q–Q plot for the difference between the paired values.

For the statistical analysis method, SDA-V2 automatically chooses the appropriate test for the selected variables based on the following criteria:

1. If two populations are independent, data in both groups are normally distributed, and variances are equal, two independent samples t-test is applied [13,15,16].
2. If two populations are independent and data in both groups are normally distributed, but variances are not equal, Welch's test is applied [17,18].
3. If two populations are independent and data in either group violates the normal assumption, the Wilcoxon rank sum test is applied [19,20].
4. If two populations are dependent and the difference between the paired values is normally distributed, the paired samples t-test is applied [13,15,16].
5. If two populations are dependent and the difference between the paired values violates the normal assumption, the Wilcoxon match-paired signed rank test is applied [14,19].

- **One-Way ANOVA**

For data exploration and data visualization, SDA-V2 automatically creates multiple boxplots for comparison and a means plot with the chosen confidence level. It also computes commonly used basic statistics within each factor level, including sample size, mean, standard deviation, minimum, maximum, range, and standard error for the responses.

For underlying assumption checking, SDA-V2 automatically performs the Shapiro–Wilk normality test to determine whether the responses within each factor level follow a normal distribution. If the responses within all factor levels are normally distributed, the application then performs the Bartlett test of homogeneity of variances.

For the statistical analysis method, SDA-V2 automatically chooses the appropriate test for selected variables based on the following criteria:

1. If the responses within each factor level are normally distributed, and variances of the responses within all factor levels are equal, one-way ANOVA is applied [21].

2. If the responses within each factor level are normally distributed, and variances of the responses within all factor levels are not equal, Welch ANOVA is applied [21, 22].

3. If the responses within either factor level violate the normal distribution assumption, the Kruskal-Wallis test is applied [23].

- **Correlation**

For data exploration and visualization, SDA-V2 automatically creates a scatter plot and correlogram. It also computes commonly used basic statistics, including sample size, mean, median, maximum, minimum, standard deviation, variance, and interquartile range for both selected variables.

For underlying assumption checking, SDA-V2 automatically performs the Shapiro–Wilk normality test to determine whether the data of the two variables follow a normal distribution.

For the statistical analysis method, SDA-V2 chooses the appropriate test for selected variables based on the following criteria:

1. If the data of both variables are normally distributed, the Pearson correlation is applied [24–29].

2. If the data of only one of the variables are normally distributed, the Spearman rank correlation is applied [27, 29].

- **Simple Regression**

For data exploration and visualization, SDA-V2 automatically generates a scatter plot with a regression line. It also calculates commonly used basic statistics, including sample size, mean, median, maximum, minimum, standard deviation, variance, and interquartile ranges for both an independent variable and a response variable.

To check underlying assumptions, SDA-V2 automatically fits a simple linear regression model and verifies assumptions related to error terms, including normality and constant variance. If any assumption concerning error terms is not met, SDA-V2 automatically performs data transformation, then fits a simple linear regression model and rechecks the assumptions.

For the statistical analysis method, SDA-V2 automatically chooses the appropriate model for selected variables based on the following criteria:

1. If error terms of the simple linear regression model from the original data exhibit normal distribution and constant variance, the simple linear regression model with the least squares method is applied.

2. If error terms of the simple linear regression model from the original data do not exhibit either normal distribution or constant variance, data transformations ($X^2, \sqrt{X}$, and $\mathrm{LOG}(X)$) are applied. The simple linear regression model is then fitted with the transformed data, and assumptions regarding error terms are checked. If all assumptions are met, the simple linear regression model with the transformed data is applied.

3. If error terms of the simple linear regression model from both the original and transformed data do not exhibit either normal distribution or constant variance, rank-based and quantile regressions are applied [30].

- **Association**

For data exploration and visualization, SDA-V2 automatically creates a clustered bar chart and mosaic plot. It also generates a contingency table with a count, Chi-square contribution, row percent, column percent, and total percent.

For underlying assumption checking, SDA-V2 determines the expected frequencies of each cell and the number of cells with expected frequencies less than 5.

For the statistical analysis method, SDA-V2 automatically chooses the appropriate test for selected variables based on the following criteria:

1. If the number of cells with expected frequencies of at least 5 is equal to or greater than 80% of the total cells, Pearson's Chi-squared test is applied [31].

2. If the number of cells with expected frequencies of at least 5 is less than 80% of the total cells, Fisher's Exact test is applied [32].

3. If both categorical variables have two levels, Pearson's Chi-squared test with Yates' continuity correction is applied [33–35].

- **Sample Size**

SDA-V2 computes the sample size associated with the test selected and automatically creates scatter plots of sample size versus the probability of type II error and power of a test. Eqs (6)-(9) are used to calculate the sample size.

Hypothesis testing for one population mean [36]

$$n = \frac{(z_{1-\alpha/2} + z_{1-\beta})^2 \sigma^2}{\epsilon^2}, \tag{6}$$

where $\epsilon = \mu - \mu_0$.

Hypothesis testing for two independent means [37]

$$n_1 = \frac{(z_{1-\alpha/2} + z_{1-\beta})^2 \left[\sigma_1^2 + \frac{\sigma_2^2}{r}\right]}{\Delta^2}, \tag{7}$$

where n_1 and n_2 are the sample size of the first and second group, respectively, and r is the projected ratio of the two sample sizes, $r = \frac{n_2}{n_1}$, and $\Delta = \mu_1 - \mu_2$.

Hypothesis testing for two dependent means [36]

$$n = \frac{(z_{1-\alpha/2} + z_{1-\beta})^2 \sigma^2}{\Delta^2}, \tag{8}$$

where $\Delta = \mu_1 - \mu_2$.

Hypothesis testing for correlation [38]

$$n = \left[\frac{z_{1-\alpha/2} + z_{1-\beta}}{0.5 \times \ln\left(\frac{1+r}{1-r}\right)}\right]^2 + 3. \tag{9}$$

Table 1 presents the summary of packages developed in SDA-V2.

Table 1: Summary of packages developed in SDA-V2.

**Packages**	**Used for**	**Procedures**
Validity/ Reliability	Assessing the quality of research instruments	The content validity of research instruments is evaluated in terms of the Item Objective Congruence (IOC) Index and four methods–Split-Half, KR-20, KR-21, and Cronbach's Alpha–are determined as internal consistency reliability.
Basic Statistics	Finding the basic statistics of a quantitative variable	The measurements of central tendency (mean, median), position (minimum, maximum, 1st quartile, 3rd quartile), and dispersion (range, standard deviation, variance, coefficient of variation, interquartile range) are calculated.
One Mean	Comparing the mean (or median) of a population with a specified constant or hypothesized value	1. If data are normally distributed, the one sample t-test is applied.<br>2. If data are not normally distributed, a Wilcoxon signed-rank test is applied.
Two Means	Comparing the means (or medians) of two independent groups and the means (or medians) of two measurements taken from the same subject	1. If two populations are independent, data in both groups are normally distributed, and variances are equal, two independent samples t-test is applied.<br>2. If two populations are independent and data in both groups are normally distributed, but variances are not equal, Welch's test is applied.<br>3. If two populations are independent and the data in either group violates the normal, the Wilcoxon rank sum test is applied.<br>4. If two populations are dependent and the difference between the paired values is normally distributed, the paired samples t-test is applied.<br>5. If two populations are dependent and the difference between the paired values violates the normal, the Wilcoxon match-paired signed rank test is applied.
One-Way ANOVA	Comparing the means (or medians) of two or more independent groups	1. If the responses within each factor level are normally distributed, and variances of the responses of all factor levels are equal, one-way ANOVA is applied.<br>2. If the responses within each factor level are normally distributed, and variances of the responses of all factor levels are not equal, Welch's ANOVA is applied.<br>3. If the responses within either factor level violate the normal distribution assumption, the Kruskal-Wallis test is applied.
Correlation	Determining the relationships between pairs of continuous variables	1. If the data of both variables are normally distributed, the Pearson correlation is applied.<br>2. If the data of only one of the variables are normally distributed, the Spearman's rank correlation is applied.
Simple Regression	Modeling and predicting the response variable	1. If error terms of the simple linear regression model from the original data exhibit normal distribution and constant variance, the simple linear regression model with the least squares method is applied.<br>2. If error terms of the simple linear regression model from the original data do not exhibit either normal distribution or constant variance, data transformations are applied. Then, simple linear regression model is fitted with the transformed data, and assumptions regarding error terms are checked. If all assumptions are met, the simple linear regression model with the transformed data is applied.<br>3. If error terms of the simple linear regression model from both original and transformed data do not exhibit either normal distribution or constant variance, rank-based and quantile regressions are applied.
Association	Determining the association between categorical variables	1. If the number of cells with expected frequencies of at least 5 is equal to or greater than 80% of the total cells, Pearson's Chi-squared test is applied.<br>2. If the number of cells with expected frequencies of at least 5 is less than 80% of the total cells, Fisher's Exact test is applied.<br>3. If both categorical variables have two levels, Pearson's Chi-squared test with Yates' continuity correction is applied.
Sample Size	Calculating the minimum sample size required for a two-sided significance test	The sample size associated with the test selected is computed.

References

[1] Rovinelli RJ, Hambleton RK. On the use of content specialists in the assessment of criterion-referenced test item validity. Tijdschrift Voor Onderwijs Research. 1977;2:49–60. Available from: https://eric.ed.gov/?id=ED121845.

[2] Turner RC, Carlson L. Indexes of Item-Objective Congruence for Multidimensional Items. International Journal of Testing. 2003;3(2):163–171. https://doi.org/10.1207/S15327574IJT0302_5.

[3] Spearman C. The proof and measurement of association between two things. By C. Spearman, 1904. The American Journal of Psychology. 1987 Fall-Winter;100(3-4):441–471. PMID: 3322052.

[4] Walker DA. A Comparison of the Spearman-Brown and Flanagan-Rulon Formulas for Split Half Reliability under Various Variance Parameter Conditions. Journal of Modern Applied Statistical Methods. 2006;5(2):443–451. Available from: http://dx.doi.org/10.22237/jmasm/1162354620.

[5] Kuder GF, Richardson MW. The theory of the estimation of test reliability. Psychometrika. 1937;2:151–160. https://doi.org/10.1007/BF02288391.

[6] Foster RC. KR20 and KR21 for Some Nondichotomous Data (It's Not Just Cronbach's Alpha). Educational and Psychological Measurement. 2021;81(6):1172–1202. https://doi.org/10.1177/0013164421992535.

[7] Cronbach LJ. Coefficient alpha and the internal structure of tests. Psychometrika. 1951;16:297–334. https://doi.org/10.1007/BF02310555.

[8] Shapiro SS, Wilk MB. An analysis of variance test for normality (complete samples). Biometrika. 1965;52(3/4):591–611. https://doi.org/10.2307/2333709.

[9] Razali NM, Wah YB. Power comparisons of Shapiro-Wilk, Kolmogorov-Smirnov, Lilliefors and Anderson-Darling tests. Journal of statistical modeling and analytics. 2011;2(1):21–33.

[10] Ghasemi A, Zahediasl S. Normality tests for statistical analysis: A guide for non-Statisticians. International Journal of Endocrinology and Metabolism. 2012 Spring;10(2):486–489. https://doi.org/10.5812/ijem.3505. PMID: 23843808.

[11] Hazelton ML. A graphical tool for assessing normality. The American Statistician. 2003;57(4):285–288. https://doi.org/10.1198/0003130032341.

[12] Schucany WR, Ng HKT. Preliminary goodness-of-fit tests for normality do not validate the one-sample Student t. Communications in Statistics-Theory and Methods. 2006;35(12):2275–2286. https://doi.org/10.1080/03610920600853308.

[13] Fisher RA. Student. Annals of Eugenics. 1939;9(1):1–9. https://doi.org/10.1111/j.1469-1809.1939.tb02192.x.

[14] Rey D, Neuhäuser M. Wilcoxon-Signed-Rank Test. In: Lovric, M. (eds) International Encyclopedia of Statistical Science. Berlin, Heidelberg: Springer; 2011. https://doi.org/10.1007/978-3-642-04898-2_616.

[15] Box JF. Guinness, Gosset, Fisher, and small samples. Statistical science. 1987;2(1):45–52. http://www.jstor.org/stable/2245613.

[16] Kalpić D, Hlupić N, Lovrić M. Student's t-Tests. In: Lovric, M. (eds) International Encyclopedia of Statistical Science. Berlin, Heidelberg: Springer; 2011. https://doi.org/10.1007/978-3-642-04898-2_641.

[17] Zimmerman DW. Some properties of preliminary tests of equality of variances in the two-sample location problem. The Journal of General Psychology. 1996;123(3):217–231. https://doi.org/10.1080/00221309.1996.9921274.

[18] Hayes AF, Cai L. Further evaluating the conditional decision rule for comparing two independent means. British Journal of Mathematical and Statistical Psychology. 2007 Nov;60(Pt 2):217–244. https://doi.org/10.1348/000711005x62576. PMID: 17971268.

[19] Wilcoxon F. Individual comparisons by ranking methods. Biometrics Bulletin. 1945;1(6):80–83. https://doi.org/10.2307/3001968.

[20] Haynes W. Wilcoxon Rank Sum Test. In: Dubitzky, W., Wolkenhauer, O., Cho, KH., Yokota, H. (eds) Encyclopedia of Systems Biology. New York, NY: Springer; 2013. https://doi.org/10.1007/978-1-4419-9863-7_1185.

[21] Delacre M, Leys C, Mora YL, Lakens D. Taking parametric assumptions seriously: Arguments for the use of Welch's F-test instead of the classical F-test in One-Way ANOVA. International Review of Social Psychology. 2019;32(1):13. https://doi.org/10.5334/irsp.198.

[22] Celik N. Welch's ANOVA: Heteroskedastic skew-t error terms. Communications in Statistics-Theory and Methods. 2022;51(9):3065–3076. https://doi.org/10.1080/03610926.2020.1788084.

[23] Kruskal WH, Wallis WA. Use of ranks in one-criterion variance analysis. Journal of the American statistical Association. 1952;47(260):583–621. https://doi.org/10.1080/01621459.1952.10483441.

[24] Binder A. Considerations of the place of assumptions in correlational analysis. American Psychologist Journal. 1959;14(8):504–510. https://doi.org/10.1037/h0048094.

[25] Kowalski CJ. On the effects of non-normality on the distribution of the sample product-moment correlation coefficient. Journal of the Royal Statistical Society. Series C (Applied Statistics). 1972;21(1):1–12. https://doi.org/10.2307/2346598.

[26] Bland JM, Altman DG. Correlation, regression, and repeated data. BMJ. 1994;308(6933):896. https://doi.org/10.1136/bmj.308.6933.896. PMID: 8173371.

[27] Headrick TC. A Note on the Relationship between the Pearson Product-Moment and the Spearman Rank-Based Coefficients of Correlation. Open Journal of Statistics. 2016;6(6):1025–1027. http://dx.doi.org/10.4236/ojs.2016.66082.

[28] Schober P, Boer C, Schwarte LA. Correlation Coefficients: Appropriate Use and Interpretation. Anesth Analg. 2018 May;126(5):1763–1768. https://doi.org/10.1213/ane.0000000000002864. PMID: 29481436.

[29] Heuvel EVD, Zhan Z. Myths About Linear and Monotonic Associations: Pearson's r, Spearman's ρ, and Kendall's τ. The American Statistician. 2022;76(1):44–52. https://doi.org/10.1080/00031305.2021.2004922.

[30] Wenz SE. What Quantile Regression Does and Doesn't Do: A Commentary on Petscher and Logan (2014). Child Development. 2019 Jul;90(4):1442–1452. https://doi.org/10.1111/cdev.13141. PMID: 30267567.

[31] Pearson K. On the criterion that a given system of deviations from the probable in the case of a correlated system of variables is such that it can be reasonably supposed to have arisen from random sampling. In: Kotz, S., Johnson, N.L. (eds) Breakthroughs in Statistics. Springer Series in Statistics. New York, NY: Springer; 1992. https://doi.org/10.1007/978-1-4612-4380-9_2.

[32] Fisher RA. The design of experiments. London: Oliver and Boyd Ltd., Edinburgh; 1935.

[33] Yates F. Contingency table involving small numbers and the χ^2 test. Supplement to the Journal of the Royal Statistical Society. 1934;1(2):217–235. https://doi.org/10.2307/2983604.

[34] Grizzle JE. The Teacher's Corner. The American Statistician. 1967;21(4):28–32. https://doi.org/10.1080/00031305.1967.10479835.

[35] Camill G, Hopkins KD. Applicability of chi-square to 2 x 2 contingency tables with small expected cell frequencies. Psychological Bulletin. 1978;85(1):163–167. https://doi.org/10.1037/0033-2909.85.1.163.

[36] Chow SC, Shao J, Wang H. Sample Size Calculations in Clinical Research. 2nd ed. Florida: Chapman and Hall/CRC; 2008.

[37] Rosner B. Fundamentals of biostatistics. 8th ed. Raleigh, NC: Cengage Learning; 2015.

[38] Hulley SB, Cummings SR, Browner WS, Grady D, Newman TB. Designing clinical research: an epidemiologic approach. Philadelphia, PA: Lippincott Williams and Wilkins; 2015.
